# Scalable psychological interventions for Syrian refugees: Preliminary results of a randomized controlled trial on the peer-refugee delivered Problem Management Plus (PM+) intervention in the Netherlands

**DOI:** 10.1192/j.eurpsy.2022.1632

**Published:** 2022-09-01

**Authors:** A. De Graaff, M. Sijbrandij, P. Cuijpers

**Affiliations:** Vrije Universiteit Amsterdam, Clinical, Neuro- And Developmental Psychology, Amsterdam, Netherlands

**Keywords:** depressive disorder, posttraumatic stress disorder, Randomized Controlled Trial, Refugees

## Abstract

**Introduction:**

In the past decade, millions of Syrians have sought refuge in neighboring countries and Europe. Refugees are at increased risk for the development of common mental disorders (CMD), such as depression and posttraumatic stress disorder (PTSD), but only a small percentage access mental health services. Problem Management Plus (PM+) is a brief, scalable intervention targeting symptoms of CMDs that can be delivered by non-specialist helpers in communities affected by adversity, such as refugees.

**Objectives:**

The aim of this randomized controlled trial (RCT) is to evaluate the effectiveness of PM+ among Syrian refugees in the Netherlands.

**Methods:**

Adult Syrian refugees and other Arabic-speaking refugees of 18 years and older with self-reported psychological distress (K10 >15) and functional impairment (WHODAS 2.0 >16) are included. Participants are randomized into PM+ or care as usual. Follow-up assessments are conducted at one-week, three-month and twelve-month follow-ups. Clinical outcomes are symptoms of depression/anxiety (HSCL-25), PTSD (PCL-5), and functional impairment (WHODAS 2.0).

**Results:**

By November 2021 [recruitment ends by December 2021], 214 participants were screened for eligibility and 184 participants were included. Participants are M=36.5yrs old (range 18-69yrs), and 73 participants are female (39.7%). We will present preliminary results for the effects of PM+ on depression, anxiety, PTSD, and functional impairment at one-week follow-up, as well as barriers and facilitators for implementing PM+ in a European country.

**Conclusions:**

After positive evaluation of peer-refugee delivered PM+, the Arabic manual and training materials will be made available through WHO to encourage scaling-up.

**Disclosure:**

No significant relationships.

